# A global analysis of CNVs in Chinese indigenous fine-wool sheep populations using whole-genome resequencing

**DOI:** 10.1186/s12864-021-07387-7

**Published:** 2021-01-23

**Authors:** Chao Yuan, Zengkui Lu, Tingting Guo, Yaojing Yue, Xijun Wang, Tianxiang Wang, Yajun Zhang, Fujun Hou, Chune Niu, Xiaopin Sun, Hongchang Zhao, Shaohua Zhu, Jianbin Liu, Bohui Yang

**Affiliations:** 1 Lanzhou Institute of Husbandry and Pharmaceutical Sciences of Chinese Academy of Agricultural Sciences, Sheep Breeding Engineering Technology Research Center, Lanzhou, 730050 China; 2 Gansu Provincial Sheep Breeding Technology Extension Station, Sunan, 734031 China; 3 Xinjiang Gongnaisi Breeding Sheep Farm, Xinyuan, 835808 China; 4 Aohan Banner Breeding Sheep Farm, Chifeng, 024300 China

**Keywords:** Copy number variation, Fine-wool sheep, Whole-genome resequencing

## Abstract

**Background:**

Copy number variation (CNV) is an important source of genetic variation that has a significant influence on phenotypic diversity, economically important traits and the evolution of livestock species. In this study, the genome-wide CNV distribution characteristics of 32 fine-wool sheep from three breeds were analyzed using resequencing.

**Results:**

A total of 1,747,604 CNVs were detected in this study, and 7228 CNV regions (CNVR) were obtained after merging overlapping CNVs; these regions accounted for 2.17% of the sheep reference genome. The average length of the CNVRs was 4307.17 bp. “Deletion” events took place more frequently than “duplication” or “both” events. The CNVRs obtained overlapped with previously reported sheep CNVRs to variable extents (4.39–55.46%). Functional enrichment analysis showed that the CNVR-harboring genes were mainly involved in sensory perception systems, nutrient metabolism processes, and growth and development processes. Furthermore, 1855 of the CNVRs were associated with 166 quantitative trait loci (QTL), including milk QTLs, carcass QTLs, and health-related QTLs, among others. In addition, the 32 fine-wool sheep were divided into horned and polled groups to analyze for the selective sweep of CNVRs, and it was found that the relaxin family peptide receptor 2 (*RXFP2*) gene was strongly influenced by selection.

**Conclusions:**

In summary, we constructed a genomic CNV map for Chinese indigenous fine-wool sheep using resequencing, thereby providing a valuable genetic variation resource for sheep genome research, which will contribute to the study of complex traits in sheep.

**Supplementary Information:**

The online version contains supplementary material available at 10.1186/s12864-021-07387-7.

## Background

Copy number variation (CNV), an important part of genomic structural variation, mainly refers to the insertion, deletion and duplication of 1 kb–5 Mb DNA fragments within the genome [1, 2]. As a type of genetic marker, CNVs extensively exist in various forms within the scope of the genome. In comparison with single nucleotide polymorphisms (SNPs), CNVs can disturb genetic expression and can exert a greater impact on the phenotype [3, 4]. Large-scale CNV detection has been carried out mainly using array comparative genome hybridization (aCGH) chips and high-density SNP chips in the past, but these methods have certain limitations, such as low coverage and low resolution, and they cannot be used to detect some new or rare CNVs. With the decline of the cost of sequencing, next generation sequencing (NGS) has overcome the limitations of chips, and shown enormous advantages for genomic CNV detection.

Numerous researches on CNV maps of livestock species such as cattle, goats, sheep and pigs have already beenreported, and the results showed that these CNVs obviously affect the production performance of livestock [5–8]. It was found that 1 kb sequence deletion in the guanylate binding protein 2 (*GBP2*) gene of cattle was significantly correlated with growth and development characteristics, indicating that CNV could serve as a marker for the molecular breeding of cattle [9]. And CNV in the endothelin receptor A (*EDNRA*) gene in goats was positively correlated with white coat coverage [10]. Additionally, the distal-less homeobox 3 (*DLX3*) gene overlapped with a CNV region (CNVR) related to wool curling, dispalying that CNV could beidentified as a candidate for the special curly wool phenotype of Tan sheep [11]. Also, a study conducted by Chen et al. found a 38.7 kb CNV existing in the methionine sulfoxide reductase B3 (*MSRB3*) gene, which significantly correlated with pig ear size [12]. However, these studies investigated livestock CNVs using chip technology, and there are relatively few reports on livestock CNVs identified using genomic resequencing. In addition, the majority of research on sheep CNVs has been focused on mutton sheep, whereas there has been almost no research on the CNVs of fine-wool sheep.

In this study, the CNVs of three Chinese fine-wool sheep breeds were analyzed using genomic resequencing. Additionally, we performed in-depth analyses on the functional of CNVs and further explored population genetics features of CNVs using selective sweep analysis. A large number of fine-wool sheep CNVs and candidate CNVRs were obtained in this study, thereby laying the foundation for determining the formation mechanisms for important economic characteristics in fine-wool sheep.

## Results

### Genome-wide detection of CNVs and CNVRs

Sequencing was performed on an Illumina HiSeq 4000 platform, producing high-quality NGS data for 32 fine-wool sheep (Additional file 1: Table S1). These reads were aligned to the sheep reference genome (Additional file 2: Table S2), with the coverage depth of each individual ranging from 28.08× (M373370) to 40.21× (M373981). This indicated that the sequencing depth was sufficient and CNV detection was possible.

**Fig. 1:**
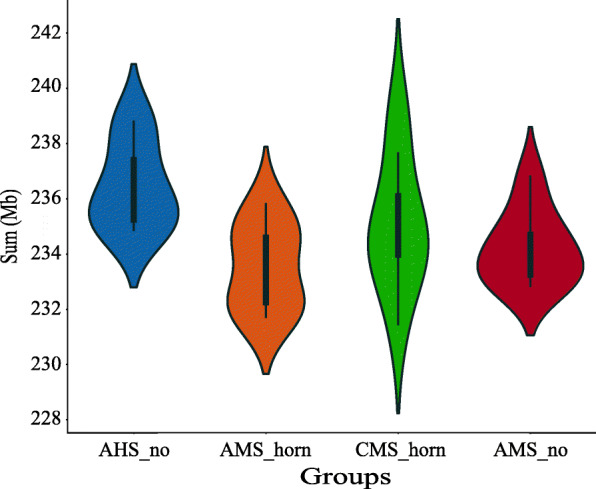
Violin plots showing distribution of the total CNV length in each group

**Fig. 2:**
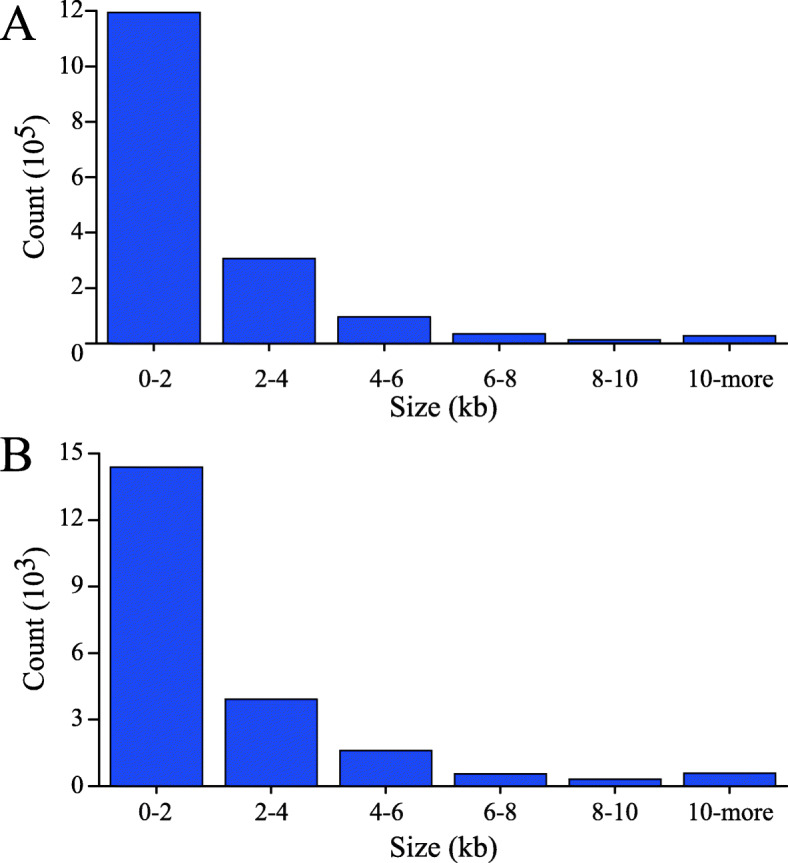
Size distribution of CNVs and CNVRs in fine-wool sheep. **a**: Size distribution of CNVs. **b**: Size distribution of CNVRs

CNVnator software, which is based on the read depth method, was utilized, and a total of 1,747,604 CNV events (including 49,851 “duplication” events and 1,697,753 “deletion” events) were detected in the 32 fine-wool sheep, with each sheep’s genome possessing 54,612.63 CNVs, on average (Table 1, Additional file 3: Table S3). To explore the CNV distribution pattern in the four groups of fine-wool sheep, violin plots were drawn for the CNV lengths. CNV lengths showed slight differences between the groups, but the total sum of CNVs from CMS_horn sheep varied widely within this population (Fig. 1). The identified CNVs ranged from 0.20 kb to 5023.60 kb in length, with an average length of 4.30 kb. The distribution showed that 69.44% of the CNVs were located within the 0–2 kb interval, 19.49% were within 2–4 kb, and 11.07% were greater than 4 kb in length (Fig. 2a).

**Table 1:** Summary of CNVs and CNVRs identified in 32 fine-wool sheep

	Breeds	Count	Duplication	Deletion	Both	Length (Mb)	Average (kb)	Precentage of chromosome by CNVRs (%)
CNVs	AMS_no	427,844	12,657	415,187	–	1874.08	4.38	–
	AMS_horn	428,669	12,545	416,124	–	1868.48	4.36	–
	CMS_horn	444,221	12,429	431,792	–	1881.06	4.23	–
	AHS_no	446,870	12,220	434,650	–	1883.50	4.21	–
CNVRs	AMS_no	5233	705	4518	10	13.5	2.58	0.52
	AMS_horn	5297	725	4567	5	14.03	2.65	0.54
	CMS_horn	5394	694	4689	11	14.14	2.62	0.55
	AHS_no	5441	698	4735	8	14.39	2.64	0.56

**Fig. 3:**
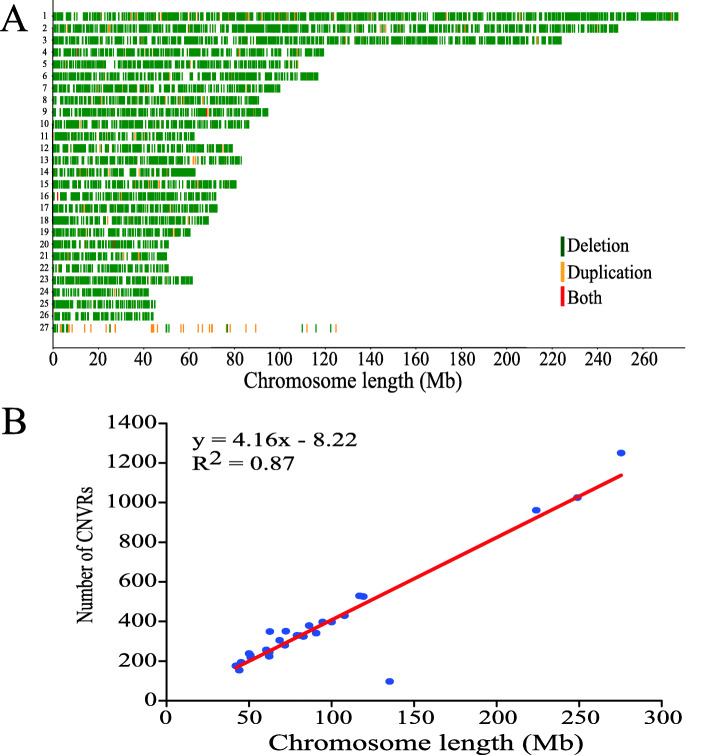
Genomic landscape of CNVRs in fine-wool sheep. **a**: A map of CNVRs in the fine-wool sheep genome; Green, orange and red represent deletion, duplication and both (deletion and duplication), respectively. **b**: Correlation between CNVR counts and chromosome length

After overlapping CNVs were merged, a total of 7228 CNVRs were obtained, with AMS_no possessing 5233, AMS_horn possessing 5297, CMS_horn possessing 5394, and AHS_no possessing 5441 (Additional file 4: Table S4, Table 1). A total of 3783 CNVRs were shared by the AMS_no, AMS_horn, CMS_horn and AHS_no sheep (Additional file 5: Fig. S1). The average length of these CNVRs was 2.62 kb, including 6345 “deletion” events, 861 “duplication” events and 22 “both” events, and the chromosome length had a significant positive linear relationship with the number of CNVRs (R^2^ = 0.87, Additional file 4: Table S4, Fig. 3). In addition, these CNVRs were nonuniformly distributed across the sheep chromosomes, with the maximum length found in *Ovis aries* chromosome one (OAR1), and the minimum found in OAR26 (Additional file 6: Fig. S2). The distribution showed that 67.35% of the CNVRs were located within the 0–2 kb interval, 18.34% were within 2–4 kb, and 14.31% were greater than 4 kb in length (Fig. 2b).

### Comparison with other studies on CNVs in sheep

The results of this study were compared with six previous reports on sheep CNVRs (Table 2). Between 111 and 3488 CNVRs have been detected in sheep in previous studies, with CNVR lengths of 10.56–120.53 Mb being reported. Between 17 and 424 of the CNVRs detected in this study overlapped with previously reported CNVRs, with overlapping ratios of 4.39–55.46%.

**Table 2:** Comparison of our study with six recent sheep CNV reports using various platforms

Study	Platform	Breed	Sample	CNVR count	CNVR length (Mb)	Overlapping CNVR count with present study	Overlapping percentage
Fontanesi et al. (2011) [7]	aCGH	6	11	135	10.56	17	12.59%
Liu et al. (2013) [13]	SNP50	3	327	238	60.35	132	55.46%
Ma et al. (2015) [14]	SNP50	8	160	111	13.76	31	27.93%
Jenkins et al. (2016) [15]	aCGH	6	30	3488	66.27	153	4.39%
Zhu et al. (2016) [16]	SNP600	3	110	490	81.04	219	44.69%
Ma et al. (2017) [11]	SNP600	1	48	1296	120.53	424	32.72%
This study	Illumina HiSeq 4000	3	32	7228	56.06	n.a.	n.a.

### Functional annotation of the identified CNVRs

To further investigate the function of these CNVRs, functional enrichment analysis of the CNVR-harboring genes was performed. A total of 119 GO terms were enriched in the CNVRs shared by the four groups of fine-wool sheep (*p* < 0.05), with these including 48 biological processes, five cellular components and 66 molecular functions (Additional file 7: Table S5). These GO terms involved sensory perception systems (GO:0007605, GO:0050954 and GO:0007600), metabolic processes (GO:0006508, GO:0043112 and GO:0055070) and growth and development processes (GO:0048610, GO:0000003 and GO:0007423), among others. According to the KEGG pathway analysis, the shared CNVR-harboring genes were enriched in 18 pathways (p < 0.05, Additional file 8: Table S6), including the Jak-STAT signaling pathway (oas04630), the Rap1 signaling pathway (oas04015), the calcium signaling pathway (oas04020), the Hippo signaling pathway (oas04390), and the estrogen signaling pathway (oas04915). Furthermore, functional enrichment analysis of the specific CNVR-harboring genes in the four groups of fine-wool sheep was also performed, and it was found that a large number of the CNVR-harboring genes participated in fat metabolism (GO:0006635, GO:0009062 and GO:0034440), amino acid metabolism (GO:0006658, GO:0006659 and GO:0005234), microelement metabolism (GO:0005506, GO:0010167 and GO:0006766), and response to stimuli (GO:0032102, GO:0032104 and GO:0009733), among other processes (Additional file 7: Table S5, Additional file 8: Table S6).

### QTLs overlapping with identified CNVRs

CNVRs detected in the four groups of fine-wool sheep were compared with a database of previously reported sheep QTLs to further analyze their hereditary effects. It was found that 1855 of the CNVRs were associated with 166 QTLs, with the QTL frequency ranging from 1 to 500. These QTLs included milk, carcass and health-related QTLs, among others, providing important information for improving fine-wool sheep in the future (Additional file 9: Table S7).

### Population genetics of CNVRs

**Fig. 4:**
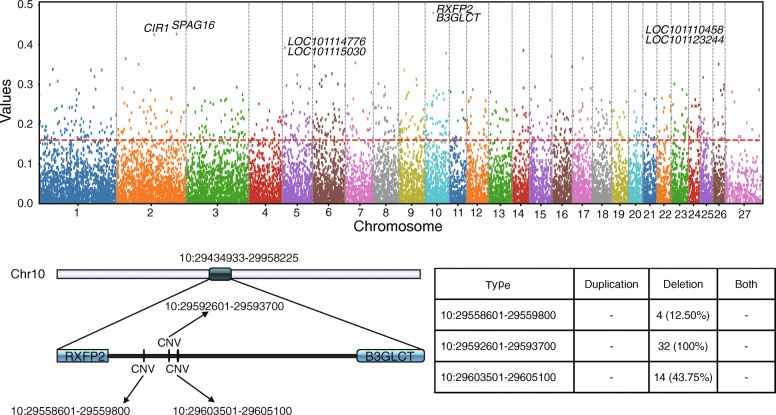
Genome wide VST value plots for CNVRs. The horizontal red dashed line represent top 5% of VST value

The 32 fine-wool sheep were divided into horned and polled groups, and selective sweep analysis of all the CNVRs was performed. As can be seen in Fig. 4 and Table S8 (Additional file 10), the horned and polled fine-wool sheep showed genetic differentiation in many of their chromosomes, with the most significant variation on chromosome 10, in the *RXFP2* and *B3GLCT* gene. Further analysis revealed that this locus contains three CNVs (10:29558601–29,559,800, 10:29592601–29,593,700, and 10:29603501–29,605,100), all of which belong to the “deletion” type. The CNVRs with the top five VST values were selected as candidate CNVRs, and the functional enrichment analysis of the genes annotated by these CNVRs was carried out. A total of 77 GO terms were found to be enriched (Additional file 11: Table S9), and they were mainly associated with fat metabolism and responses to stress. In addition, seven KEGG pathways were enriched (Additional file 12: Table S10), including olfactory transduction, the Notch signaling pathway, and the renin-angiotensin system, among others.

### qPCR validation of CNVRs

To confirm the accuracy of our CNVR predictions, we randomly selected 10 CNVRs in 12 sheep samples to validate via qPCR. As shown in Fig. S3 (Additional file 13), eight (80%) of the randomly selected CNVRs were confirmed in agreement using CNVnator software.

## Discussion

In this study, NGS technology was used to detect the CNVs in 32 indigenous fine-wool sheep in China. A total of 1,747,604 CNV events were detected, with each sheep, on average, possessing 54,612.63 CNVs. In comparison with previous CNV detection methods based on SNP chips and aCGH, NGS has many advantages for the determination of both the number and size of CNVs [7, 14]. With its high sensitivity for CNV detection, NGS can identify CNV boundaries more accurately [17]. A total of 7228 CNVRs were obtained after merging overlapping CNVs, which greatly exceeded the numbers previously reported for sheep based on SNP50 chip and SNP600 chip studies [11, 13, 14, 16]. This difference was not surprising, as the genomic coverage of SNP chips is poor, which results in the detection of longer CNVRs [18, 19]. The CNVRs detected in this study accounted for 2.17% of the sheep reference genome, which falls within the range (0.8–5.12%) reported for horses, pigs, cattle and chickens [20–23]. However, the CNVRs identified in individual species accounted for more than 10% of their reference genomes, which may be related to the different genetic backgrounds of the studied animals [24, 25]. Studies have shown that the number of CNVRs detected in populations consisting of a variety of species may be higher than the numbers detected in populations only containing a single species [19]. In addition, these results could also be ascribed to differences in the CNV calling algorithms and standards used to determine the CNVs [26, 27]. Therefore, further development of bioinformatics algorithms and tools to generate high reliability CNVs is necessary for improving the quality of CNV studies. In the CNVs identified in this study, “deletion” events were far more frequent than “duplication” events, which concurred with the similar disequilibrium phenomenon found in studies of other species [8, 28]. This may be because of the higher sensitivity of CNV calling algorithms to deletion events, as it is easier to identify a missing segment of the genome than an amplified one when there are limited numbers of sequence reads [21].

Keeping in mind that the detection rate of CNVRs is affected by many factors, the results of this study were compared with those of six previous studies on sheep CNVs. The CNVRs identified in these previous studies were different to some extent, which may have been related to the differences in sheep breeds, sample sizes, CNV detection platforms and CNV calling algorithms used. However, it is noteworthy that the CNVRs identified in this study had high overlapping ratios (27.93–55.46%) with the CNVRs identified by Liu et al., Ma et al., Zhu et al., and Ma et al., but had low overlapping ratios (4.39–12.59%) with the CNVRs detected by Fontanesi et al., and Jenkins et al., [7, 11, 13–16]. The four studies with which there were high overlapping ratios all used Chinese indigenous sheep breeds or Chinese cultivated sheep breeds as the study subjects, whereas the two studies with which there were low overlapping ratios used foreign sheep breeds. It was also noted that when comparing to studies using the Illumina OvineSNP BeadChip to detect sheep CNVs, the number of CNVRs overlapping with those identified in this study tended to increase as the number of probes on the chip increased from SNP50 to SNP600. The use of different CNV calling algorithms also has a substantial effect on the results of CNVR studies. The software packages currently commonly used for CNV detection include PennCNV, CNVcaller, and CNVnator. PennCNV software has been extensively applied to Illumina chip data, especially for high-density SNP data [16, 29]. CNVcaller and CNVnator software use read depth methods to detect CNVs in resequencing data [30, 31].

In this study, many of the CNVR-harboring genes were significantly enriched for GO terms relating to sensory perception. This concurred with the results of a study on the CNVs in humans, yak, pigs, horses, dogs and mice, which also found that GO terms relating to sensory perception were significantly enriched [32–37]. A previous study also found that, in comparison with cattle, gene families related to sensory perception were significantly enriched in yak [38]. Yak generally live in alpine pastoral areas which have serious shortages of fodder grasses in spring and winter, and a well-developed sensory perception system could improve their ability to acquire food. The three fine-wool sheep breeds used in this study are mainly farmed in extensive grazing systems, and their sensory perception-related gene families may have therefore rapidly expanded to adapt to the environment and its shortages of fodder grasses, and alpine and drought environmental pressures. Many GO terms related to substance metabolism were also enriched, and these GO terms were also related to the environment in which the fine-wool sheep selected for this study were located. Fine-wool sheep live in an extremely harsh environment, so substance metabolism mechanisms are of great importance for their production and reproduction. In addition, Wnt-related signaling pathways were also enriched in some of the CNVR-harboring genes in the AMS_no group. Studies in humans and mice have shown that Wnt signaling plays a crucial role in hair follicle development and hair growth during the transition from the resting period to the growth period [39, 40]. The three sheep breeds selected for this study are mainly used for wool production. Furthermore, AMS wool quality is superior to that of CMS_horn and AHS_no [41–43]. Therefore, the Wnt signaling pathway may make an important contribution to the hair follicle development process in AMS.

Through the analysis of KEGG signaling pathways, it was found that some of the CNVR-harboring genes were enriched for signaling pathways correlated with wool growth and development. It has been reported that, as one of the important pathways in the follicle development process, the Jak-STAT signaling pathway can stimulate MAPK to influence follicle development [44]. The skin is the largest non-genital organ targeted by estrogens, which can significantly change the cyclic response of the hair follicles. Estrogens can lengthen the hair growing period and shorten the rest period, thereby promoting rapid hair regeneration [45, 46]. In addition, some signaling pathways related to microelement and vitamin metabolism were also enriched. A shortage of microelements and vitamins can influence wool growth by influencing follicle development [47].

Many studies have shown that CNVRs contain QTLs associated with important economic traits in animals [48, 49]. Therefore, the CNVRs detected in this study were compared with the QTLs reported in the sheep QTL database. The QTL categories found in this study were basically identical to those found in pigs and cattle. The health-related QTLs found included fecal egg count QTLs, worm count QTLs and worm length QTLs. Previous studies have reported that worm disease infection rates in sheep can exceed 70% in many countries, causing huge losses to the livestock industry [50, 51]. Relative to barn-fed livestock, gazing livestock are more likely to be infected with worms. These results indicate that CNVs, which are a critical type of genetic variation, may have an important effect on sheep health.

We divided the 32 sheep into horned and polled groups for CNVR selective sweep analysis, to investigate the genetic role of CNVs in fine-wool sheep horn type domestication processes. The *RXFP2* gene was found to be intensely selected between the two groups. Many previous studies have confirmed that *RXFP2* is the main candidate gene related to sheep horn type [52–55]. Some genes associated with physical features in sheep are artificially selected in a directional manner during the domestication process. CNVs may therefore accumulate in sheep populations under these selection pressures, thereby forming the genetic basis for important economic characteristics.

## Conclusions

In this study, the first resequencing-based CNV map of Chinese indigenous fine-wool sheep was developed, providing an important addition to the previously published sheep CNVs. This information will be beneficial for future investigations of the genomic structural variations underlying traits of interest in sheep.

## Methods

### Animal and sample collection

We collected blood samples from 32 fine-wool sheep (2-year-old rams, Additional file 14: Table S11), including 16 Alpine Merino sheep (8 horned, AMS_horn; 8 polled, AMS_no, Gansu Provincial Sheep Breeding Technology Extension Station), eight Chinese Merino sheep (horned, CMS_horn, Xinjiang Gongnaisi Breeding Sheep Farm) and eight Aohan fine-wool sheep (polled, AHS_no, Aohan Banner Breeding Sheep Farm), respectively, and the animals were released after the sample collection. Blood samples were collected using the jugular vein blood sampling method, and were preserved in EDTA anti-freezing tubes at − 20 °C.

### Construction of sequencing library and sequencing

Genomic DNA was extracted from the blood using a TIANamp Genomic DNA Kit according to the manufacturer’s instructions. The integrity and purity of the DNA was determined using 1.5% agarose gel electrophoresis and a NanoDrop 2000. The DNA concentration was measured using a Qubit 2.0. Aliquots (1.5 μl) of DNA were taken from each sample, and library construction was performed according to the Truseq Nano DNA HT instructions. Briefly, ultrasonic waves were used to fragment the DNA into 350 bp sections, after which end repair was performed, and A tails and DNA fragment connectors were added; finally, the PCR end-products were purified. Agilent 2100 and real-time PCR were used to conduct quality tests for fragment size and concentration on the constructed library. All the libraries were sequenced using the Illumina HiSeq 4000 platform, 150 bp of paired-end reads were generated, and the insert size was approximately 350 bp.

### Raw data preprocessing and alignment

The raw data were generated by Illumina sequencing. Low-quality reads, linkers, and primers were removed using Trimmomatic software (v0.32) to obtain clean reads, with the parameters set as MINLEN = 50, LEADING = 20, TRAILING = 20, and SLIDINGWINDOW = 5,20 [56]. The clean reads were aligned to the sheep reference genome (Oar_v4.0, GCF_000298735.2) by BWA software (v0.7.11), using the following alignment parameters: mem -t 4 -k 32 –M [57]. Repetitions were removed from the alignment results using the rmdup command in SAMTOOLS software [58]; if multiple read pairs had identical external coordinates, only the pair with the highest mapping quality was retained.

### CNV calling and CNVR determination

CNVnator software (version 0.3) was used to detect the CNVs in the sheep genome samples [31]. After setting the sliding window to a fixed value of 100 bp, the CNVs were detected according to the standards recommended by Abyzov et al. [31]. CNVs with *p*-values less than 0.01 (e-val1 calculated using t-test statistics), sizes greater than 1 kb, and fractions of mapped reads with zero quality (q0) less than 0.5 were filtered and used for downstream analysis. The gene copy numbers (CN) of the genomes were estimated using the CNVnator “-genotype” option. CNVRs were determined by aggregating overlapping CNVs identified in different individuals, as described by Redon et al. [25], who classified them as “deleted” (CN < 0.4), “conserved” (0.4 ≤ CN ≤ 1.6), or “duplicated” (CN > 1.6).

### Comparison with previous studies

We compared our results with the reported results of another sheep CNV study, to verify the reliability of our study. Because the previous study used the Oar_v3.1 reference genome, NCBI Genome Remapping software (https://www.ncbi.nlm.nih.gov/genome/tools/remap) was used to convert Oar_v3.1 feature annotation locations to the Oar_v4.0 reference genome. We then took these intersections according to our CNVR interval position information and counted the number and lengths of all CNVRs.

### Functional enrichment analysis of CNVR-harboring genes

BioMart software (http://www.biomart.org/) was used to retrieve the CNVR-harboring genes from the Ensembl database, and completely and partially (≥50%) overlapped genes were reserved for later analysis. The DAVID database (http://david.abcc.ncifcrf.gov/) was used for the gene ontology (GO) and Kyoto Encyclopedia of Genes and Genomes (KEGG) functional enrichment analyses [59], and p-values ≤0.05 were considered to indicate significant enrichment in the candidate gene. In addition, to study whether these CNVRs were associated with economically important characteristics in fine-wool sheep, these CNVRs were compared with QTLs in the sheep QTL database (https://www.animalgenome.org/cgi-bin/QTLdb/OA/index, sheep QTL database).

### Population genetics of CNVRs

The principles of CNV selective sweep analysis are similar to that of SNP selective sweep analysis. The selective sweep analysis of the CNVs in this study was carried out according to Redon’s method [25]. The VST matrix is similar to Fst (highly correlated with Wright’s fixation index F_ST_), in which the data used for calculating population differences are based on copy number. VST is calculated at the SNP level by considering (VT-VS)/VT, where VT is the variance in LRRs (log-R ratios) of SNPs (within a defined CNVR) estimated among individuals of two populations, and VS is the average variance within each breed, weighted for breed size (in our case: horned versus polled). Positive VST values indicate that the CNVRs have a high level of population difference with respect to a wide range of genomes, and this may relate to selection. We averaged SNP VST values within a given CNVR to obtain a mean VST value for each CNVR. Subsequently, the CNVRs with the top five VST values were taken as candidate CNVRs, and functional enrichment analysis of these regions was performed.

### qPCR validation of CNVRs

Ten CNVRs were randomly selected, and quantitative PCR (qPCR) was used for verification in 12 sheep. According to its location, we obtained the sequence of each CNVR fragment from the UCSC Bioinformatics Website, and we used Primer 5.0 software to design specific primers for those fragments (Additional file 15: Table S12). Genomic DNA samples that were used for resequencing were also used for qPCR verification. Following a previous report, *DGAT2* was used as the reference gene [60]. We use TransStart Green qPCR SuperMix and a LightCycler 480 II instrument for qPCR, and each sample was run in triplicate to ensure quantification accuracy. We used the 2^-ΔΔCt^ method to calculate the multiple of change, and we calculated 2 × 2^-ΔΔCt^ for the copy number of the target gene in the test sample [16].

## Supplementary Information

**Additional file 1: Table S1.** Number of reads and bases in quality control.

**Additional file 2: Table S2.** Read mapping statistics and coverage of depth.

**Additional file 3: Table S3.** List of all CNV events in each individual.

**Additional file 4: Table S4.** Detail information of the detected CNVRs.

**Additional file 5: Figure S1.** Venn diagram of CNVR numbers in four different fine-wool sheep groups.

**Additional file 6: Figure S2.** CNVRs length for 27 chromosomes across four different fine-wool sheep groups.

**Additional file 7: Table S5.** Functional enrichment of GO analysis of CNVR-harbored genes.

**Additional file 8: Table S6.** Functional enrichment of KEGG analysis of CNVR-harbored genes.

**Additional file 9: Table S7.** Information about QTLs overlapping with identified CNVRs.

**Additional file 10: Table S8.** List of VST values and annotated genes.

**Additional file 11: Table S9.** GO analysis of CNVR-harbored genes differentially expressed between the horned and polled groups.

**Additional file 12: Table S10.** KEGG analysis of CNVR-harbored genes differentially expressed between the horned and polled groups.

**Additional file 13: Figure S3.** qPCR validation of selected CNVRs.

**Additional file 14: Table S11.** Introduction of the 3 sheep breeds examined in the present study.

**Additional file 15: Table S12.** Primers used in this study for qPCR.

## Data Availability

All the whole genome sequencing raw data used in this study has been deposited in the Sequence Read Archive (SRA) public databases under BioProject (PRJNA680869).

## Abbreviations

CNV: Copy number variation
CNVR: CNV region
QTL: Quantitative trait loci
SNP: Single nucleotide polymorphism
aCGH: Array comparative genome hybridization
NGS: Next generation sequencing
AMS: Alpine Merino sheep
CMS_horn: Chinese Merino sheep
AHS_no: Aohan fine-wool sheep
GO: Gene ontology
KEGG: Kyoto Encyclopaedia of Genes and Genomes
