# Supplementary figures and images for: A global analysis of CNVs in Chinese indigenous fine-wool sheep populations using whole-genome resequencing
Source: BMC Genomics. 2021 Jan 23;22:78. doi: 10.1186/s12864-021-07387-7 (PMC7825165; doi:10.1186/s12864-021-07387-7)

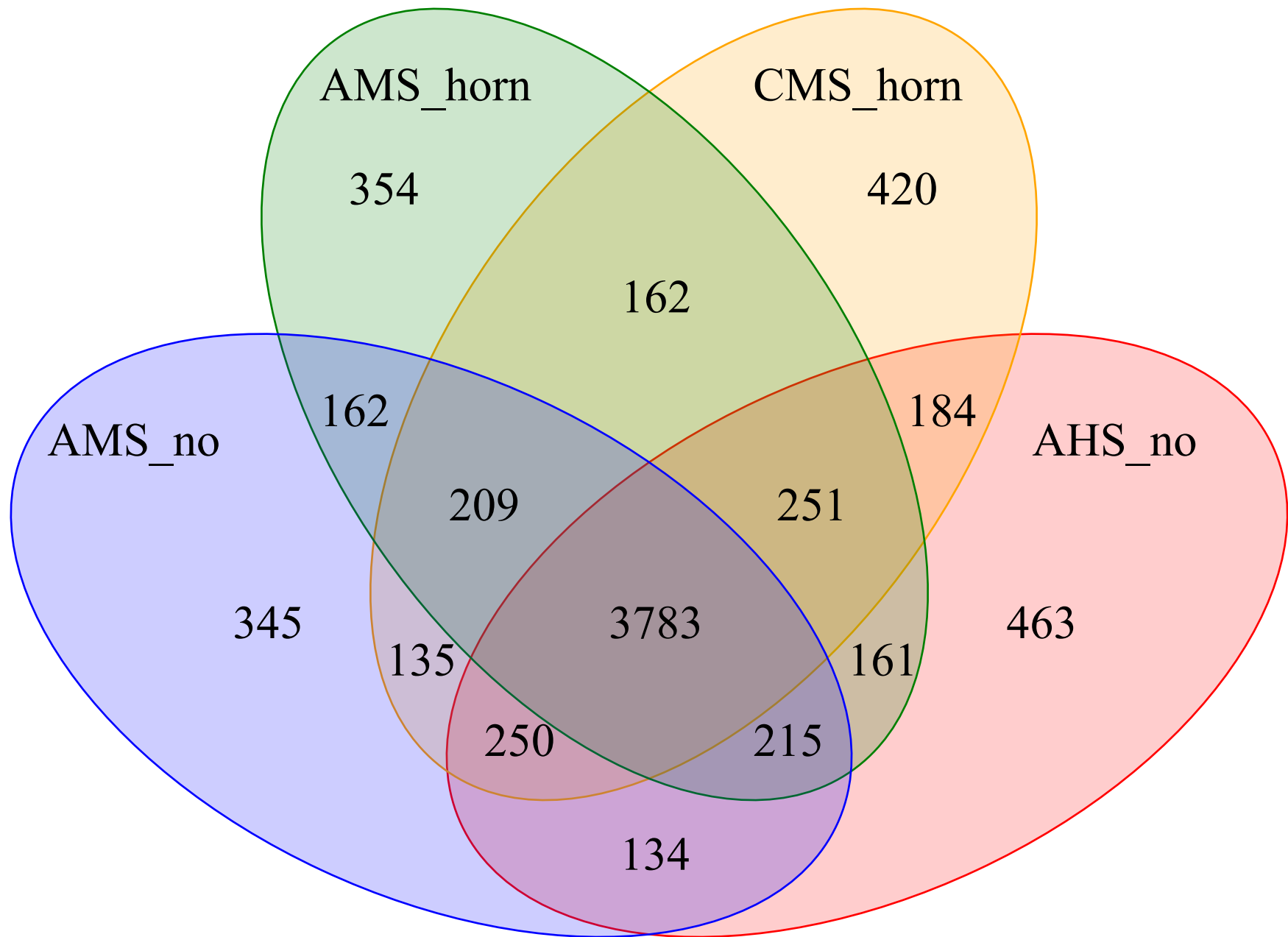

Supplement: Supplementary file 5 — Additional file 5: Figure S1. Venn diagram of CNVR numbers in four different fine-wool sheep groups. [file 12864_2021_7387_MOESM5_ESM.pdf]

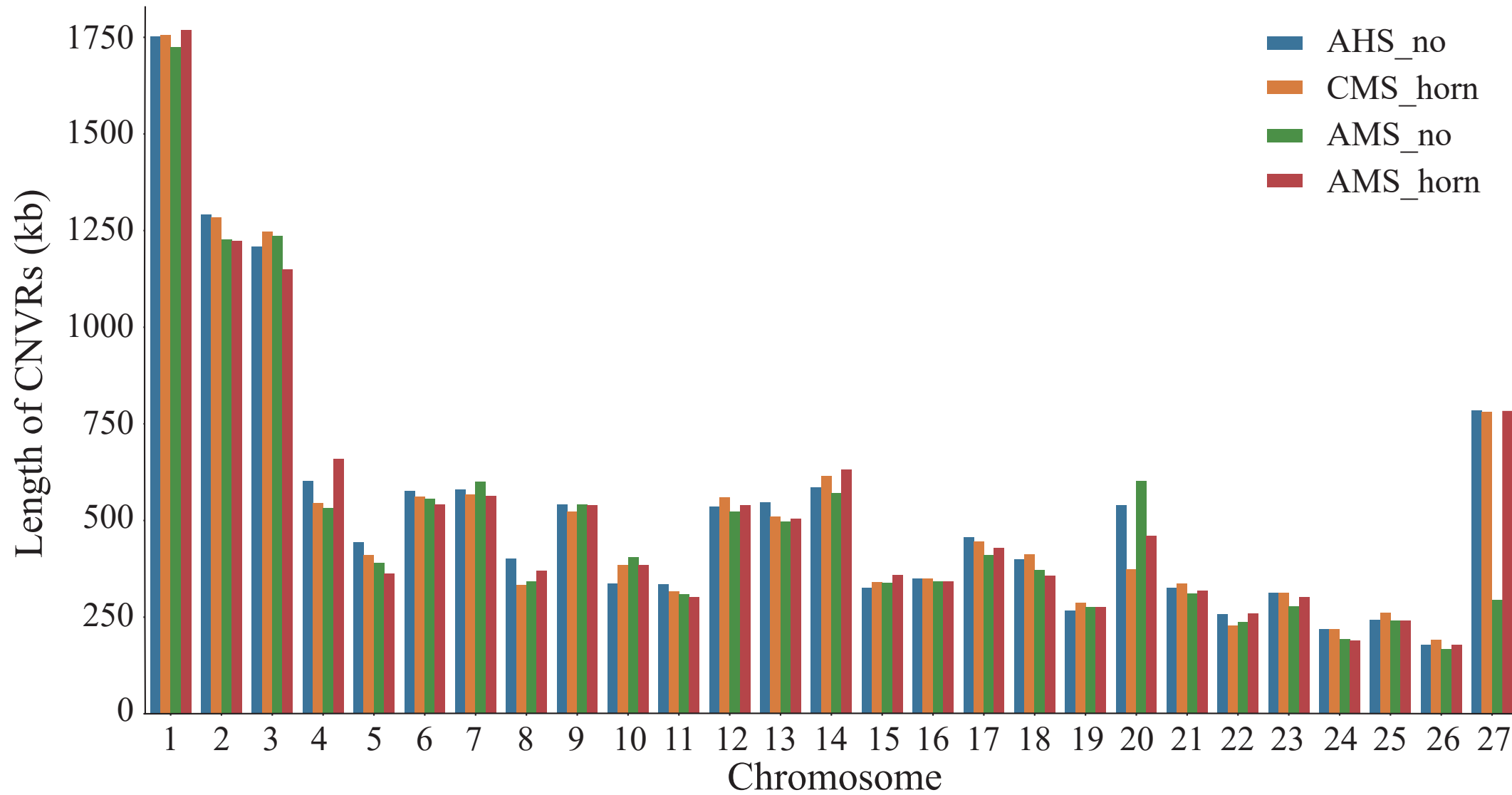

Supplement: Supplementary file 6 — Additional file 6: Figure S2. CNVRs length for 27 chromosomes across four different fine-wool sheep groups. [file 12864_2021_7387_MOESM6_ESM.pdf]

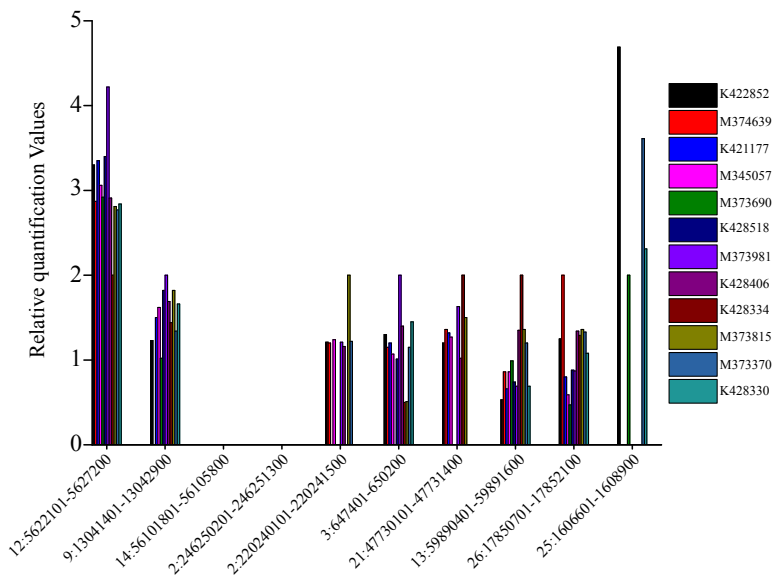

Supplement: Supplementary file 13 — Additional file 13: Figure S3. qPCR validation of selected CNVRs. [file 12864_2021_7387_MOESM13_ESM.pdf]
